# Association of Acute Kidney Injury with the Risk of Dementia: A Meta-Analysis

**DOI:** 10.3390/jcm10194390

**Published:** 2021-09-26

**Authors:** Salman Hussain, Ambrish Singh, Benny Antony, Rolando Claure-Del Granado, Jitka Klugarová, Radim Líčeník, Miloslav Klugar

**Affiliations:** 1 Czech National Centre for Evidence-Based Healthcare and Knowledge Translation (Czech EBHC: JBI Centre of Excellence, Masaryk University GRADE Centre Cochrane, Czech Republic), Institute of Biostatistics and Analyses, Faculty of Medicine, Masaryk University, Kamenice 5, 625 00 Brno, Czech Republic; klugarova@med.muni.cz (J.K.); radim.licenik@gmail.com (R.L.); klugar@med.muni.cz (M.K.); 2 Menzies Institute for Medical Research, University of Tasmania, 17 Liverpool Street, Hobart, TAS 7000, Australia; ambrish.singh@utas.edu.au (A.S.); benny.eathakkattuantony@utas.edu.au (B.A.); 3 Division of Nephrology, Hospital Obrero No 2–CNS, Cochabamba, Bolivia; rclaure@yahoo.com; 4 Universidad Mayor de San Simon School of Medicine, Cochabamba, Bolivia

**Keywords:** acute kidney injury, dementia, dialysis, epidemiology, systematic review, meta-analysis

## Abstract

Acute kidney injury (AKI) is associated with several adverse outcomes, including new or progressive chronic kidney disease, end-stage kidney disease, and mortality. Epidemiological studies have reported an association between AKI and dementia as a long-term adverse outcome. This meta-analysis was aimed to understand the association between AKI and dementia risk. A literature search was performed in MEDLINE and Embase databases, from inception to July 2021, to identify epidemiological studies reporting the association between AKI and dementia risk. Title and abstract followed by the full-text of retrieved articles were screened, data were extracted, and quality was assessed, using the Newcastle–Ottawa scale by two investigators independently. The primary outcome was to compute the pooled risk of dementia in AKI patients. Subgroup analysis was also performed based on age and co-morbidities. Certainty of evidence was assessed using the GRADE approach. Statistical analysis was performed using Review Manager 5.4 software. Four studies (cohort (*n* = 3) and case–control (*n* = 1)) with a total of 429,211 patients, of which 211,749 had AKI, were identified. The mean age of the patients and the follow-up period were 64.15 ± 16.09 years and 8.9 years, respectively. Included studies were of moderate to high quality. The pooled estimate revealed a significantly higher risk of dementia in AKI patients with an overall relative risk/risk ratio (RR) of 1.92 (95% CI: 1.52–2.43), *p* ≤ 0.00001. Dementia risk increases by 10% with one year increase in age with an RR of 1.10 (95% CI: 1.09–1.11), *p* < 0.00001. Subgroup analysis based on stroke as a co-morbid condition also revealed significantly higher dementia risk in AKI patients (RR 2.30 (95% CI: 1.62–3.28), *p* = 0.009). All-cause mortality risk was also significantly higher in AKI patients with dementia with a pooled RR of 2.11 (95% CI: 1.20–3.70), *p* = 0.009. The strength of the evidence was of very low certainty as per the GRADE assessment. Patients with AKI have a higher risk of dementia. Further large epidemiological studies are needed to confirm the mechanistic association.

## 1. Introduction

Acute kidney injury (AKI) is a complex disorder characterized by an abrupt decline in kidney function over a short period of time [1]. AKI is associated with poor quality of life, decreased productivity, and adverse health economic impact [2,3]. The reported prevalence of AKI ranged from 1 to 66%, with a varied incidence between high-income and low-to-middle-income countries [3,4]. Many recent epidemiologic studies have shown that patients with AKI are at a higher risk of developing chronic kidney disease (CKD), end-stage kidney disease (ESKD), cardiovascular diseases, and acute neurological complications such as attention deficits, decreased mental status, seizures, and hyperreflexia [5,6,7,8,9]. Dementia is a neurodegenerative disorder characterized by progressive deterioration of intellectual function, and it is one of the leading causes of limiting the capacity for independent living for the elderly population [10,11]. Over the last few decades, the global prevalence of dementia has increased considerably [12]. It is important to identify the determinants of dementia, particularly in the absence of effective treatments [13]. Numerous studies have provided evidence on the associations between various modifiable risk factors and cognitive decline or dementia later in life [14,15]. Both AKI and dementia are significant public health concerns and are associated with poor health outcomes and rising health care costs for society [3]. The occurrence of dementia in patients with AKI is of clinical importance as dementia is associated with an increased humanistic and economic burden [12].

Studies have reported that chronic kidney conditions such as CKD and ESKD are associated with accelerated cardiovascular events and share common risk factors with dementia [16,17,18,19]. These common vascular co-morbidities such as hypertension, diabetes mellitus, or hyperlipidemia, may also have a role in the development of dementia in this population [20]. Few previous meta-analyses found the increased odds for cognitive impairment in patients with CKD and renal dysfunction [18,21]. In addition, an animal study has found that AKI-induced inflammation adversely impacts the brain, among other organs [5]. The brain and kidneys share similar hemodynamic and anatomic pathways. Vascular damage due to alteration in the blood–brain barrier, high vascular permeabilities, and inflammatory cascades could be the potential mechanism for the occurrence of dementia in AKI patients [22,23].

Although the long-term neurological effects of AKI are unclear, these factors may predispose patients with AKI to an increased risk of developing dementia, as observed in a few recent studies [24,25]. Although primary epidemiological studies exploring this association are limited, the risk of dementia in the AKI population can be assessed using meta-analytical techniques to pool the evidence from real-world data studies.

The preliminary search of existing systematic reviews or meta-analysis was performed on July 2021 in Epistemonikos, PROSPERO, Open Science Framework, Cochrane Library, and JBI Evidence Synthesis, and no reviews evaluating the association of acute kidney injury with the risk of dementia were identified. Hence, we conducted a meta-analysis of existing evidence from primary epidemiological studies that compared the risk of dementia in patients with AKI versus individuals without AKI.

## 2. Materials and Methods

### 2.1. Protocol

The present meta-analysis followed the preferred reporting items for systematic review and meta-analysis (PRISMA) and Meta-analysis of Observational Studies in Epidemiology (MOOSE) reporting guidelines [26,27]. Refer to Table S1 and Table S2 for the checklists. The protocol of the current study was prospectively published as a preprint at medRxiv [28].

### 2.2. Search Strategy

A three-step search strategy was utilized to locate both published and unpublished studies. An initial, limited search was undertaken in MEDLINE (Ovid) using keywords and index terms related to AKI and dementia. An analysis of the text words in the title and abstract as well as the index terms used to describe the articles were followed. A second search using all identified keywords and index terms was conducted in MEDLINE (Ovid) and Embase (Ovid) databases (the search period was from inception to 14 July 2021). Thirdly, the reference lists of all studies that met the inclusion criteria were checked manually for additional records. Lastly, abstract booklets of major international nephrology and neurology congress—World Congress of Nephrology, American Society of Nephrology, European Renal Association–European Dialysis and Transplant Association (ERA-EDTA), Asian Pacific Congress of Nephrology, Neuroscience, Alzheimer’s Association International Conference (AAIC), and American Academy of Neurology—from the last two years were also searched. Citation tracking was also performed for all the articles qualified for inclusion. The search strategy used in this study is available in Table S3. The literature search was not restricted to any date or language; however, only the studies published in English were included.

### 2.3. Study Selection/Inclusion Criteria

Studies that are eligible for inclusion into the meta-analysis must be observational analytical studies (prospective, retrospective, cohort, or case–control) that assessed the risk of dementia in the AKI population compared to the risk of dementia in the population without AKI. Primary studies including individuals with AKI with dementia at the entry of the cohort were excluded from the analysis. Eligible studies must report relative risk/risk ratio (RR), hazard ratio (HR), or odds ratio (OR) with 95% confidence intervals (CI) or must provide enough raw data to calculate those ratios. In the case of insufficient information, primary study authors were contacted.

The studies retrieved from the database search were evaluated against eligibility for inclusion using the Covidence software (Covidence systematic review software, Veritas Health Innovation, Melbourne, Australia. Available at www.covidence.org, accessed on 20 September 2021) by two investigators (S.H. and A.S.) independently, firstly by title/abstract screening and secondly by full-text screening. Studies excluded from full-text screening are available in Table S4 with reasons for exclusion. In the case of discrepancies in the inclusion of a study, the agreement was reached by consensus and/or by consulting the third investigator (M.K.).

### 2.4. Data Extraction

An excel-based, standardized data collection form was used to extract the information: study title, first author, year of publication, country/countries where the study was conducted, study population, methods used to identify control/cohort, methods used to confirm the diagnosis of AKI and dementia, number of cases and control/cohort size, demographics of the cases and control/cohort, the average duration of follow-up, confounders that were adjusted for, and the adjusted effect estimates with 95% CI.

The data extraction was independently performed, in duplicate, by two investigators (S.H. and A.S.), ensuring the accuracy of the data extracted. The extracted data for all studies were then cross-checked by the third investigator (M.K.) for any data discrepancies which were resolved by referring to the primary source.

### 2.5. Quality Assessment and Certainty of the Evidence

We used Newcastle–Ottawa (NOS) quality assessment scale to evaluate the quality of the included studies. Two reviewers assessed the quality of eligible studies independently. The NOS is a standard quality assessment tool used to evaluate the quality of the observational study on the basis of three domains: (1) the recruitment of the cases and controls, (2) the comparability between cases and controls, and (3) the ascertainment of the key outcomes of interest [29]. Based on the score achieved by the individual study, a high, medium, or low quality of the study was determined. Studies were not excluded from meta-analyses based on the quality assessment; however, the influence of the quality on the results of meta-analyses was explored by the sensitivity analyses.

The Grading of Recommendations Assessment, Development and Evaluation (GRADE) tool was used to assess the certainty or quality of the evidence [30]. The GRADE working group rated the certainty of evidence as high, moderate, low, or very low certainty of evidence based on the study design, risk of bias, inconsistency, indirectness, imprecision, and other considerations.

### 2.6. Statistical Analysis

We used Cochrane’s (London, UK) Review Manager 5.4 data analysis software to perform the meta-analysis. The dementia events in AKI patients are considered as rare; therefore, odds ratio, RR, and hazard ratio were used interchangeably. For simplicity, RR was used for all these measures [31]. We used the generic inverse-variance method (GIVM) to combine the point estimates from each study to calculate pooled effect estimates. The GIVM of the DerSimonian and Laird assigns the weight for each study in the pooled analysis in reverse to its variance.

Considering the high probability of between-study variance due to distinction in populations and techniques used to diagnose AKI and dementia, the random-effect model was picked over the fixed-effect model. We used the Cochran’s Q test, complemented with the I2 statistic, to evaluate the between-study statistical heterogeneity [32]. The I2 statistic quantifies the proportion of total variation across studies resulting from heterogeneity rather than chance. The value of I2 = 0–25% represents insignificant heterogeneity, 25–50% low heterogeneity, 50–75% moderate heterogeneity, and more than 75% high heterogeneity [33,34]. Subgroup analysis based on age, co-morbidities, and the all-cause mortality rate was performed. Sensitivity analysis was performed using the leave-one-out method to assess if pooled effect estimates were influenced by any single study alone or by the risk of bias in the included studies. Summary of findings tables were created using the GRADEpro GDT tool [35].

## 3. Results

### 3.1. Studies Characteristics

**Figure 1:**
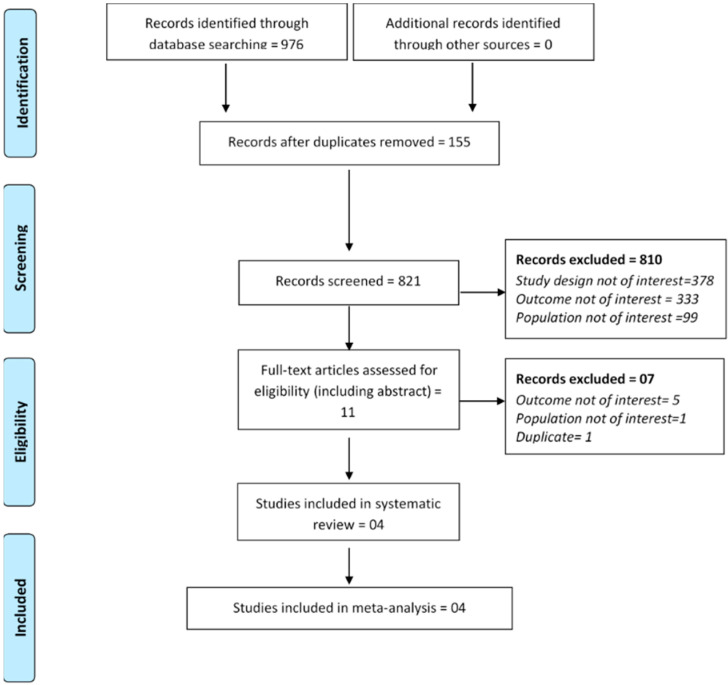
PRISMA flowchart showing study inclusion process.

Of 976 citations retrieved, four articles [24,25,36,37], including one abstract [37], qualified for inclusion in this meta-analysis with a total of 429,211 patients, of which 211,749 had AKI. PRISMA diagram showed the detailed study inclusion process (Figure 1).

The mean age of the patients and the follow-up period were 64.15 ± 16.09 years and 8.9 years, respectively. All the included studies were retrospective cohort in nature, except the study by Wu et al. [37], which was a case–control study. Studies were conducted in Taiwan (*n* = 2), China (*n* = 1), and the USA (*n* = 1) and published between 2017 and 2020. There were two studies from Taiwan [25,36]; Tsai et al., used the national health insurance research database (NHIRD), and Kao et al. used the longitudinal health insurance database (LHID). The study from the US [24] used clinical and administrative data from intermountain healthcare—an organization that covers patients from the Utah and Idaho region. AKI was ascertained based on the International Classification of Diseases (ICD) 9th edition codes, procedure codes, and definitions outlined in KDIGO guidelines, while dementia was confirmed using ICD-9 codes and clinical modification codes in all the included studies (Table 1).

### 3.2. Quality Assessment and Certainty of the Evidence

Based on NOS for non-randomized studies, the methodological quality of included studies was moderate to high quality with a mean score of 8 (range: 6–9). However, the inherent bias of observational studies design should be considered while interpreting the result. Refer to Table 2 for a detailed quality assessment.

The evidence on the association between AKI and risk of dementia was of very low certainty as per the GRADE rating system. Certainty assessment ratings and the summary of findings are presented in Table 3.

### 3.3. Meta-Analysis

**Figure 2:**
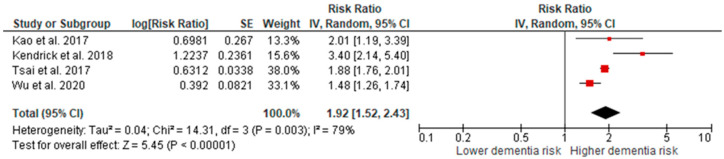
Meta-analysis showing the pooled risk of dementia in AKI patients compared to patients without AKI.

The pooled estimate revealed a significantly higher risk of dementia in AKI patients compared to patients without AKI with an overall RR of 1.92 (95% CI: 1.52–2.43), *p* ≤ 0.00001 (Figure 2).

**Figure 3:**
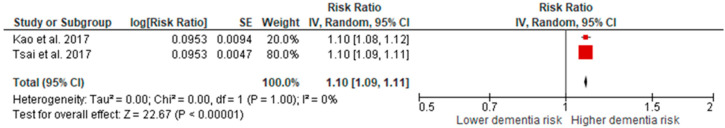
Risk of dementia in AKI patients with one year increase in age.

This pooled estimate was based on adjusted RR (adjusted for all possible confounding factors such as age, sex, previous cognitive dysfunction, and several co-morbidities including diabetes, hypertension, hyperlipidemia, head injury, depression, stroke, chronic obstructive pulmonary disease, estimated glomerular filtration rate, coronary artery disease, congestive heart failure, atrial fibrillation, cancer, liver disease, and chronic infection/inflammation). Dementia risk increases 10% with one year increase in age with an RR of 1.10 (95% CI: 1.09–1.11), *p* < 0.00001 (Figure 3).

**Figure 4:**
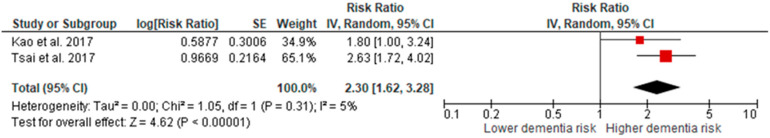
Risk of dementia in AKI patients with stroke.

Subgroup analysis based on stroke as a co-morbidity revealed significantly higher dementia risk (Figure 4) in AKI patients (RR 2.30 (95% CI: 1.62 to 3.28), *p* ≤ 0.00001).

**Figure 5:**
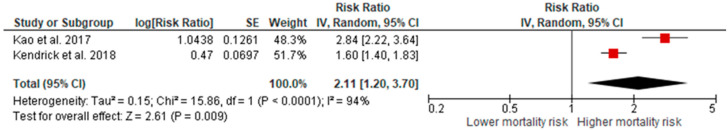
All-cause mortality risk in AKI patients with dementia.

All-cause mortality risk was also significantly higher (Figure 5) in AKI patients with dementia than patients without AKI with a pooled RR of 2.11 (95% CI: 1.20–3.70), *p* = 0.009. Only one study analyzed and reported a higher dementia risk with a hazard ratio of 2.01 (95% CI: 1.19–3.39), *p* = 0.01 in AKI patients who survived at least 90 days after recovery from acute dialysis.

### 3.4. Sensitivity Analysis

There was no evidence of change in significance level of effect size as confirmed through sensitivity analysis by omitting each study one by one (leave-one-out) from the pooled analysis. Refer to Table S5 for the sensitivity plot.

## 4. Discussion

This is the first meta-analysis to investigate the association of AKI with dementia risk. A significantly higher dementia risk was observed in patients with AKI as compared to patients without AKI in an adjusted analysis (adjusted for several possible confounding factors). Sensitivity analysis also revealed a consistently higher risk of dementia in AKI patients. All-cause mortality risk was higher in AKI patients who developed dementia.

CKD is a common risk factor for the development of dementia, and CKD following AKI might be responsible for the development of dementia. AKI increases the risk of developing new or progressive CKD (HR: 2.67) and ESKD (HR: 4.67) [38]. The studies by Tsai et al. [25] and Kao et al. [36] suggest that there may be other pathways also to develop dementia in AKI patients apart from CKD only. Furthermore, there is considerable overlap in the pathophysiology, risk factors, and outcomes between AKI and CKD [39,40]. A plethora of evidence found a higher risk of developing dementia in patients with co-morbidities [41,42,43]. AKI patients with co-morbidities (diabetes, hypertension, depression, chronic obstructive pulmonary disease, coronary artery disease, congestive heart failure, atrial fibrillation, malnutrition, and inflammation) were also found to have significantly higher dementia risk [42,43]. Due to the reversible nature of AKI, long-term outcomes remain ambiguous and debatable.

Cognitive impairment is a well-recognized complication of CKD [18]. Evidence from the literature found a worsening of cognitive function as the kidney function declines [44,45,46]. A recent cohort study using clinical practice research datalink found a co-occurrence of CKD and dementia in the real-world setting [47]. There is inconclusive research on the mechanistic association of AKI with the development of dementia. Evidence from preclinical studies suggested that AKI influences the blood–brain barrier permeability and may be responsible for various brain and hippocampal complications [48,49]. Hippocampal involvement in AKI patients could be due to the upregulation of macrophage scavenger receptor 1, serum amyloid A3, Ras homolog gene family member J, downregulation of G protein-coupled receptor 34 and 124, and others [50]. Due to dysregulation of transporters across the blood–brain barrier, AKI causes an imbalance of excitatory and inhibitory neurotransmitters [51]. According to the evidence from the clinical study, it could be assumed that AKI is an independent risk factor for CKD development and is associated with multiple organ dysfunction [52]. The kidneys and the brain share similar vascoregulatory and anatomic pathways [53]. The kidneys and the brain are more susceptible to vascular damage due to a high amount of blood flow. This damage can alter endothelial dysfunction, which leads to loss of vascoregulatory abilities [54]. This can affect the brain through inflammatory cascades generated via oxidative stress, proapoptotic pathways activation, and may lead to dementia [22,23].

Age is also a potential risk factor for the development of dementia, as we have observed in our meta-analysis that the risk of dementia increases 10% with one year increase in age. Kao et al. [36], in the extended analysis (adjusted for age and potential confounders), found an increased risk of dementia in AKI patients after the age of 58 years compared to non-AKI patients. Higher dementia risk was observed in AKI patients with stroke as a co-morbidity. Evidence from a recent meta-analysis confirms stroke as an independent and potentially modifiable risk factor based on the analysis of 1,885,536 participants [55]. In our meta-analysis, the all-cause mortality rate was higher in AKI patients with dementia. High mortality in AKI patients is not only attributable to renal failure but also to extra renal complications and its impact on other distant organs [56]. A Danish national registry-based cohort study concluded dementia as an independent risk factor for all-cause mortality, and the mortality rate further exceeds with co-morbidities [57]. In addition to that, another study from the same registry found a higher annual mortality rate ratio in patients with dementia aged ≥65 years in the last 20 years (1996–2015) [58].

The strength of the current study includes a literature search in major databases, an extensive search of major nephrology and neurology conference proceedings, and citation tracking of all the included articles. The majority of the included studies were of high-quality with a large sample size and use of the GRADE approach to rate the certainty of evidence. Pooled analysis was based on data adjusted for several possible confounding factors, which strengthen the conclusion. Furthermore, subgroup and sensitivity analysis also confirm the higher dementia risk.

This meta-analysis has few limitations, such as a diagnosis of AKI based on ICD-9 codes in the majority of the included studies, and dementia was also identified based on ICD-9 codes. Classification of disease based on ICD-9 codes may lead to misdiagnosis as the included studies collected data retrospectively. Preexisting cognitive impairment was not ruled out at the baseline of the study in two of the included studies and might have imparted influence on the overall results; however, a higher risk of developing dementia risk was consistent in the sensitivity analysis. We noticed significantly high heterogeneity among the included studies. In order to explore the heterogeneity, a random effect model was chosen, and findings from subgroup analysis were reported separately. Lastly, retrospective studies are typically a source of inherent bias, which decreased the certainty of the evidence.

Overall, this meta-analysis showed a higher dementia risk in AKI patients. Future studies should look to establish the mechanistic association of AKI with dementia and stratification of dementia risk as per AKI stages. Furthermore, future, large prospective studies should adjust the findings for potential clinical (patients with non-recovery/partial recovery vs. complete recovery) and other associated covariates.

## 5. Conclusions

In conclusion, this meta-analysis demonstrated a significantly higher risk of dementia among patients with AKI compared with individuals without AKI. The strength of evidence was of very low certainty as per the GRADE assessment. Further large epidemiological studies are needed to confirm the mechanistic association. We recommend close monitoring of patients for dementia after AKI to curtail morbidity.

## Figures and Tables

**Table 1:** Characteristics of included studies.

Author, Year, and Country	Study Design	Database Used	Study Duration	Follow-Up Period	Cohort Size	AKI Patients	Non-AKI Patients	Mean Age (Years)	Female (%)	Assessment of AKI	Assessment of Outcomes (Dementia)	Number of Dementia Cases in AKI/Non-AKI Group	Unadjusted Hazard Ratio/Risk Ratio	Adjusted Hazard/Risk Ratio/Odds Ratio	Study Adjusted for
Kao et al. 2017; Taiwan [36]	Cohort	Longitudinal Health Insurance Database	1999–2008	NR	3445	689	2756	63.33 ± 16.19	41.90%	Procedure code	ICD-9-CM codes (290.X, 290.XX, 294.X, 294.XX, 331.X)	44/67	NR	2.01 (95% CI: 1.19–3.39)	Adjusted for baseline co-morbidities, acute organ dysfunction, and the propensity score
Kendrick et al. 2019, USA [24]	Cohort	Intermountain Healthcare	1999–2009	5.8 years	2082	1041	1041	61 ± 16	NR	ICD-9 codes and KDIGO guidelines	ICD-9 codes (290 to 290.4 and 331)	73/24	NR	3.4 (95% CI: 2.14–5.40); composite outcome of dementia or death: 1.60 (1.40, 1.84)	Propensity matched
Tsai et al. 2017; Taiwan [25]	Cohort	Taiwan’s National Health Insurance Research Database	2000–2011	12 years	415576	207788	207788	68.13 ± 16.08	39.20%	(ICD-9-CM Code 584	ICD9-CM Codes 290, 294.1, 331.0	3265/4806	NR	1.88 (95% CI: 1.76–2.01)	Study adjusted for age, sex, and several co-morbidities (diabetes, hypertension, hyperlipidemia, head injury, depression, stroke, chronic obstructive pulmonary disease, coronary artery disease, congestive heart failure, atrial fibrillation, cancer, liver disease, chronic infection/inflammation, autoimmune disease, malnutrition
Wu et al. 2020 * [37]	Case–control	NR	NR	NA	8108	2231	5877	NR	NR	KDIGO guidelines	NR	NR	NR	1.48 (95% CI: 1.26–1.74)	Adjusted for estimated glomerular filtration rate, age, albumin level, hypertension, myocardial infarction, congestive heart failure, peripheral vascular disease, cerebrovascular disease, chronic lung disease, connective tissue disease, moderate/severe renal disease, tumor, and anemia

AKI: Acute Kidney Injury; ICD-9: International Classification of Disease, 9th Edition; KDIGO: Kidney Disease Improving Global Outcomes; NA: Not Applicable; NR: Not Reported; U.S: United States of America. * Represents conference abstract.

**Table 2:** Quality assessment of included studies.

**Cohort Studies**	**Selection**				**Comparability**	**Outcome**			
Study Author	Representation of the Exposed Cohort	Selection of the Non-Exposed Cohort	Ascertainment of Exposure	Demonstration that Outcome of Interest Was Not Present at the Start of the Study	Comparability of Cohorts on the Basis of Design or Analysis	Assessment of Outcome	Was Follow-Up Long Enough for Outcomes to Occur	Accuracy of Follow-Up of Cohorts	Overall Score
Kao, 2017, Taiwan [36]	✓	✓	✓	✓	✓✓	✓	✗	✓	High (8)
Kendrick, 2019, USA [24]	✓	✓	✓	✓	✓✓	✓	✓	✓	High (9)
Tsai, 2017, Taiwan [25]	✓	✓	✓	✓	✓✓	✓	✓	✓	High (9)
**Case-Control Study**	**Selection**				**Comparability**	**Outcome**			
Study author	Is the case definition adequate	Representativeness of the Cases	Selection of Controls	Definition of Controls	Comparability of Cases and Controls on the Basis of the Design or Analysis	Ascertainment of Exposure	Same method of ascertainment for cases and controls	Non-Response Rate	Overall Score
Wu, 2020, China [37]	✓	✗	✓	✓	✓✓	✗	✗	✓	Medium (6)

✓: Yes; ✗: No.

**Table 3:** Summary of findings table showing certainty of evidence for dementia risk in AKI compared to non-AKI patients. **Patients:** Dementia risk in AKI patients compared to non-AKI patients. **Risk factor**: Dementia incidence. **Comparisons**: Non-AKI patients.

Certainty Assessment							№. of Patients		Effect		Certainty	Importance
№ of Studies	Study Design	Risk of Bias	Inconsistency	Indirectness	Imprecision	Other Considerations	AKI and Dementia Risk	Placebo	Relative (95% CI)	Absolute (95% CI)		
Dementia Risk												
4	observational studies	not serious	serious ^a^	not serious	not serious	none	3382/211749 (1.6%)	4897/217462 (2.3%)	RR 1.92 (1.52 to 2.43)	21 more per 1000 (from 12 more to 32 more)	⨁ ◯ ◯ ◯ VERY LOW	High importance

**CI:** Confidence interval; **RR:** Risk ratio. Explanations: a. Presence of significantly high heterogeneity (I^2^ = 79%). **GRADE Working Group grades of evidence:** Very low certainty: The true effect is probably markedly different from the estimated effect.

## Data Availability

The data that support the findings of this study are available from the corresponding author upon reasonable request.

## Supplementary Materials

The following are available online at https://www.mdpi.com/article/10.3390/jcm10194390/s1, Table S1: PRISMA checklist; Table S2: MOOSE checklist; Table S3: Complete search strategy; Table S4: List of articles excluded with reasons during full-text screening; Table S5: Sensitivity plot.

## Conference Presentation

Abstract from this work was presented by the first author (Salman Hussain) in the form of an oral presentation at the 3rd AKI CRRT Congress 2019 held at Kuala Lumpur, Malaysia. The presenter received a meritorious award for this oral presentation.
