# Supplementary material for: Association of Acute Kidney Injury with the Risk of Dementia: A Meta-Analysis
Source: J Clin Med. 2021 Sep 26;10(19):4390. doi: 10.3390/jcm10194390 (PMC8509598; doi:10.3390/jcm10194390)
Supplement: Supplementary file 1 [file jcm-10-04390-s001.zip › jcm-1386504-SI.pdf]

Supplementary Table S1 **(PRISMA 2020 Checklist)**

Section and Topic	Item #	Checklist item	Location where item is reported
**TITLE**			
Title	1	Identify the report as a systematic review.	1
**ABSTRACT**			
Abstract	2	See the PRISMA 2020 for Abstracts checklist.	1
**INTRODUCTION**			
Rationale	3	Describe the rationale for the review in the context of existing knowledge.	2
Objectives	4	Provide an explicit statement of the objective(s) or question(s) the review addresses.	2
**METHODS**			
Eligibility criteria	5	Specify the inclusion and exclusion criteria for the review and how studies were grouped for the syntheses.	3
Information sources	6	Specify all databases, registers, websites, organisations, reference lists and other sources searched or consulted to identify studies. Specify the date when each source was last searched or consulted.	3
Search strategy	7	Present the full search strategies for all databases, registers and websites, including any filters and limits used.	Supplement
Selection process	8	Specify the methods used to decide whether a study met the inclusion criteria of the review, including how many reviewers screened each record and each report retrieved, whether they worked independently, and if applicable, details of automation tools used in the process.	3
Data collection process	9	Specify the methods used to collect data from reports, including how many reviewers collected data from each report, whether they worked independently, any processes for obtaining or confirming data from study investigators, and if applicable, details of automation tools used in the process.	3
Data items	10a	List and define all outcomes for which data were sought. Specify whether all results that were compatible with each outcome domain in each study were sought (e.g. for all measures, time points, analyses), and if not, the methods used to decide which results to collect.	3
	10b	List and define all other variables for which data were sought (e.g. participant and intervention characteristics, funding sources).	3

Section and Topic	Item #	Checklist item	Location where item is reported
		Describe any assumptions made about any missing or unclear information.	
Study risk of bias assessment	11	Specify the methods used to assess risk of bias in the included studies, including details of the tool(s) used, how many reviewers assessed each study and whether they worked independently, and if applicable, details of automation tools used in the process.	3
Effect measures	12	Specify for each outcome the effect measure(s) (e.g. risk ratio, mean difference) used in the synthesis or presentation of results.	4
Synthesis methods	13a	Describe the processes used to decide which studies were eligible for each synthesis (e.g. tabulating the study intervention characteristics and comparing against the planned groups for each synthesis (item #5)).	3
	13b	Describe any methods required to prepare the data for presentation or synthesis, such as handling of missing summary statistics, or data conversions.	4
	13c	Describe any methods used to tabulate or visually display results of individual studies and syntheses.	4
	13d	Describe any methods used to synthesize results and provide a rationale for the choice(s). If meta-analysis was performed, describe the model(s), method(s) to identify the presence and extent of statistical heterogeneity, and software package(s) used.	4
	13e	Describe any methods used to explore possible causes of heterogeneity among study results (e.g. subgroup analysis, meta-regression).	4
	13f	Describe any sensitivity analyses conducted to assess robustness of the synthesized results.	4
Reporting bias assessment	14	Describe any methods used to assess risk of bias due to missing results in a synthesis (arising from reporting biases).	NA
Certainty assessment	15	Describe any methods used to assess certainty (or confidence) in the body of evidence for an outcome.	4
**RESULTS**			
Study selection	16a	Describe the results of the search and selection process, from the number of records identified in the search to the number of studies included in the review, ideally using a flow diagram.	4, Fig.1
	16b	Cite studies that might appear to meet the inclusion criteria, but which were excluded, and explain why they were excluded.	NA
Study characteristics	17	Cite each included study and present its characteristics.	5, Table 1
Risk of bias in	18	Present assessments of risk of bias for each included study.	5, Table 2

Section and Topic	Item #	Checklist item	Location where item is reported
studies			
Results of individual studies	19	For all outcomes, present, for each study: (a) summary statistics for each group (where appropriate) and (b) an effect estimate and its precision (e.g. confidence/credible interval), ideally using structured tables or plots.	11-12, Fig.2-5
Results of syntheses	20a	For each synthesis, briefly summarise the characteristics and risk of bias among contributing studies.	9
	20b	Present results of all statistical syntheses conducted. If meta-analysis was done, present for each the summary estimate and its precision (e.g. confidence/credible interval) and measures of statistical heterogeneity. If comparing groups, describe the direction of the effect.	11-12
	20c	Present results of all investigations of possible causes of heterogeneity among study results.	11-12
	20d	Present results of all sensitivity analyses conducted to assess the robustness of the synthesized results.	12, supplement
Reporting biases	21	Present assessments of risk of bias due to missing results (arising from reporting biases) for each synthesis assessed.	NA
Certainty of evidence	22	Present assessments of certainty (or confidence) in the body of evidence for each outcome assessed.	10, Table 3
**DISCUSSION**			
Discussion	23a	Provide a general interpretation of the results in the context of other evidence.	12
	23b	Discuss any limitations of the evidence included in the review.	12-13
	23c	Discuss any limitations of the review processes used.	13
	23d	Discuss implications of the results for practice, policy, and future research.	13
**OTHER INFORMATION**			
Registration and protocol	24a	Provide registration information for the review, including register name and registration number, or state that the review was not registered.	4
	24b	Indicate where the review protocol can be accessed, or state that a protocol was not prepared.	medRxiv
	24c	Describe and explain any amendments to information provided at registration or in the protocol.	NA

Section and Topic	Item #	Checklist item	Location where item is reported
Support	25	Describe sources of financial or non-financial support for the review, and the role of the funders or sponsors in the review.	14
Competing interests	26	Declare any competing interests of review authors.	14
Availability of data, code and other materials	27	Report which of the following are publicly available and where they can be found: template data collection forms; data extracted from included studies; data used for all analyses; analytic code; any other materials used in the review.	14

From: Page MJ, McKenzie JE, Bossuyt PM, Boutron I, Hoffmann TC, Mulrow CD, et al. The PRISMA 2020 statement: an updated guideline for reporting systematic reviews. BMJ 2021;372:n71. doi: 10.1136/bmj.n71

Supplementary Table S2 **(MOOSE Checklist)**

Item No	Recommendation	Reported on Page No
Reporting of background should include		
1	Problem definition	1
2	Hypothesis statement	2
3	Description of study outcome(s)	2
4	Type of exposure or intervention used	2
5	Type of study designs used	2-3
6	Study population	2-3
Reporting of search strategy should include		
7	Qualifications of searchers (eg, librarians and investigators)	2
8	Search strategy, including time period included in the synthesis and key words	2-3, supplement
9	Effort to include all available studies, including contact with authors	3
10	Databases and registries searched	3
11	Search software used, name and version, including special features used (eg, explosion)	3-4
12	Use of hand searching (eg, reference lists of obtained articles)	3
13	List of citations located and those excluded, including justification	3, supplement
14	Method of addressing articles published in languages other than English	NA
15	Method of handling abstracts and unpublished studies	3
16	Description of any contact with authors	Not Required
Reporting of methods should include		
17	Description of relevance or appropriateness of studies assembled for assessing the hypothesis to be tested	3-4
18	Rationale for the selection and coding of data (eg, sound clinical principles or convenience)	3
19	Documentation of how data were classified and coded (eg, multiple raters, blinding and interrater reliability)	3
20	Assessment of confounding (eg, comparability of cases and controls in studies where appropriate)	3-4
21	Assessment of study quality, including blinding of quality assessors, stratification or regression on possible predictors of study results	3
22	Assessment of heterogeneity	4
23	Description of statistical methods (eg, complete description of fixed or random effects models, justification of whether the chosen models account for predictors of study results, dose-response models, or cumulative meta-analysis) in sufficient detail to be replicated	4
24	Provision of appropriate tables and graphics	4
Reporting of results should include		

25	Graphic summarizing individual study estimates and overall estimate	11-12, Fig.2-5
26	Table giving descriptive information for each study included	5, Table 1
27	Results of sensitivity testing (eg, subgroup analysis)	6, supplement
28	Indication of statistical uncertainty of findings	6, Table 3

Reporting of discussion should include		
29	Quantitative assessment of bias (eg, publication bias)	NA
30	Justification for exclusion (eg, exclusion of non-English language citations)	NA
31	Assessment of quality of included studies	Table 3
Reporting of conclusions should include		
32	Consideration of alternative explanations for observed results	14
33	Generalization of the conclusions (ie, appropriate for the data presented and within the domain of the literature review)	14
34	Guidelines for future research	14
35	Disclosure of funding source	14

From: Stroup DF, Berlin JA, Morton SC, et al, for the Meta-analysis Of Observational Studies in Epidemiology (MOOSE) Group. Meta-analysis of Observational Studes in Epidemiology. A Proposal for Reporting. *JAMA*. 2000;283(15):2008-2012. doi: 10.1001/jama.283.15.2008.

Supplementary Table S3 **(Search Strategy)**

MEDLINE(R) ALL <1946 to July 14, 2021> (Ovid)

Embase <1974 to 2021 July 12>
Search was conducted on 14th July 2021 at 7:50 am (CET).

#	search string	# of results
**1**	acute kidney failure/	143540
**2**	exp acute kidney injury/	145024
**3**	(acute kidney failure or acute renal failure).tw.	56643
**4**	(acute kidney injur$ or acute renal injur$).tw.	74050
**5**	(acute kidney insufficie$ or acute renal insufficie$).tw.	3404
**6**	acute tubular necrosis.tw.	8266
**7**	(ARI or AKI or ARF or AKF or ATN).tw.	82155
**8**	1 or 2 or 3 or 4 or 5 or 6 or 7	218851
**9**	exp dementia/	560482
**10**	dementia.mp.	352646
**11**	exp senile dementia/	104542
**12**	vascular dementia.mp.	17567
**13**	alzheimer dementia.mp.	1735
**14**	alzheimer disease/	314144
**15**	alzheimer* disease.mp.	416420
**16**	alzheimer type dementia.mp.	1664
**17**	9 or 10 or 11 or 12 or 13 or 14 or 15 or 16	712386
**18**	8 and 17	976

Supplementary Table S4 **(List of excluded articles)**

**Author, year**	**Title**	**Reason for exclusion**
Lanca et al. 2017 [1]	Renal outcome of diabetic versus nondiabetic patients with acute kidney injury	Outcome not of interest
Wonnacott et al. 2014 [2]	Epidemiology and outcomes in community-acquired versus hospital-acquired aki	Outcome not of interest
ThanAl-Aly et al. 2012 [3]	Greater variability in kidney function is associated with an increased risk of death	Population not of interest
Reis et al. 2019 [4]	Acute kidney injury at admission in an internal medicine department: What happens after discharge?	Outcome not of interest
Kendrick et al. 2018 [5]	AKI is associated with an increased risk of dementia	Duplicate (conference abstract)
Couchoud et al. 2018 [6]	Outcomes of acute kidney injury depend on initial clinical features: A national french cohort study	Outcome not of interest
Wu et al. 2020 [7]	Preexisting dementia is associated with increased risks of mortality and morbidity following major surgery: A nationwide propensity score matching study	Outcome not of interest

Supplementary Table S5: **Sensitivity analysis for acute kidney injury and dementia risk (omitting each study from pooled meta-analysis)**

**Study removed**	**Effect size, RR (95%CI)**
Wu et al. 2020	1.48 (1.26, 1.74)
Tsai et al. 2017	1.88 (1.76, 2.01)
Kendrick et al. 2018	3.40 (2.14, 5.40)
Kao et al. 2017	2.01 (1.19, 3.39)
**Overall effect estimate**	1.92 (1.52, 2.43)

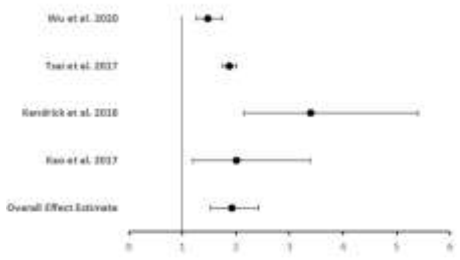

References:

1. Lanca A, Assis R, Gama P, Cortes C, Paredes S, Ferrer F, Lobos A. RENAL OUTCOME OF DIABETIC VERSUS NONDIABETIC PATIENTS WITH ACUTE KIDNEY INJURY: MP278. Nephrology Dialysis Transplantation. 2017;32.
2. Wonnacott A, Meran S, Amphlett B, Talabani B, Phillips A. Epidemiology and outcomes in community-acquired versus hospital-acquired AKI. Clinical Journal of the American Society of Nephrology. 2014;9(6):1007-14.
3. Al-Aly Z, Balasubramanian S, McDonald JR, Scherrer JF, O'hare AM. Greater variability in kidney function is associated with an increased risk of death. Kidney international. 2012;82(11):1208-14.
4. Reis M, Salvador P, Gomes AM, Ventura A, Fernandes JC. SP223 ACUTE KIDNEY INJURY AT ADMISSION IN AN INTERNAL MEDICINE DEPARTMENT: WHAT HAPPENS AFTER DISCHARGE?. Nephrology Dialysis Transplantation. 2019 Jun 1;34(Supplement_1):gfz103-SP223.
5. Kendrick J, Holmen, You Z, Chonchol M, Jovanovich A. AKI is associated with an increased risk of dementia. Journal of American Society of Nephrology. 2018;32.
6. Couchoud C, Riffaut N, Hannedouche T, Moranne O, Hertig A. Outcomes of acute kidney injury depend on initial clinical features: A national French cohort study. Revue d'Épidémiologie et de Santé Publique. 2018;66:S385.
7. Wu YM, Kuo HC, Li CC, Wu HL, Chen JT, Cherng YG, et al. Preexisting dementia is associated with increased risks of mortality and morbidity following major surgery: a nationwide propensity score matching study. International journal of environmental research and public health. 2020;17(22):8431.
